# Immunotherapy-Related Sialadenitis

**DOI:** 10.7759/cureus.80720

**Published:** 2025-03-17

**Authors:** Matthew Baron

**Affiliations:** 1 Internal Medicine, Moffitt Cancer Center, Tampa, USA

**Keywords:** immune-related adverse event (irae), immunotherapy, ipilimumab, nivolumab, sialadenitis

## Abstract

Sialadenitis is an inflammation of one or more of the salivary glands. A 21-year-old genetic female undergoing combination ipilimumab/nivolumab therapy for metastatic melanoma presented to our hospital with anterior neck swelling. CT imaging demonstrated bilateral submandibular gland sialadenitis. The clinical picture was most consistent with immunotherapy-related sialadenitis, and the patient responded promptly to systemic corticosteroids.

## Introduction

Acute sialadenitis is a relatively common malady usually arising from infection or salivary duct obstruction. Herein, we review a unique case of acute bilateral sialadenitis in the setting of nivolumab/ipilimumab combination therapy for metastatic melanoma. Nivolumab is a monoclonal antibody that binds to the programmed death 1 receptor (PD-1), and ipilimumab is a monoclonal antibody that binds to the cytotoxic T-lymphocyte associated antigen 4 (CTLA-4), thereby inhibiting the down regulation of T-cell activation and proliferation [1]. These pharmaceuticals fall under the broader umbrella of immune checkpoint inhibitors and have demonstrated efficacy in an ever-broadening array of cancers since the introduction of pembrolizumab in 2014 [2]. 

## Case presentation

A 21-year-old genetic female with a history of metastatic melanoma presented to the emergency room of our tertiary care hospital with complaints of progressive neck swelling resulting in odynophagia after initiation of combination ipilimumab/nivolumab therapy four days prior to presentation. She denied having fever, chills, dyspnea, cough, nausea, vomiting, arthralgias, myalgias, dry eye, or rash. Physical exam was notable for tender cervical adenopathy. The remainder of the physical exam, including vital signs, was within normal limits without evidence of xerostomia, dental caries, oropharyngeal erythema, or systemic lymphadenopathy.

**Figure 1:**
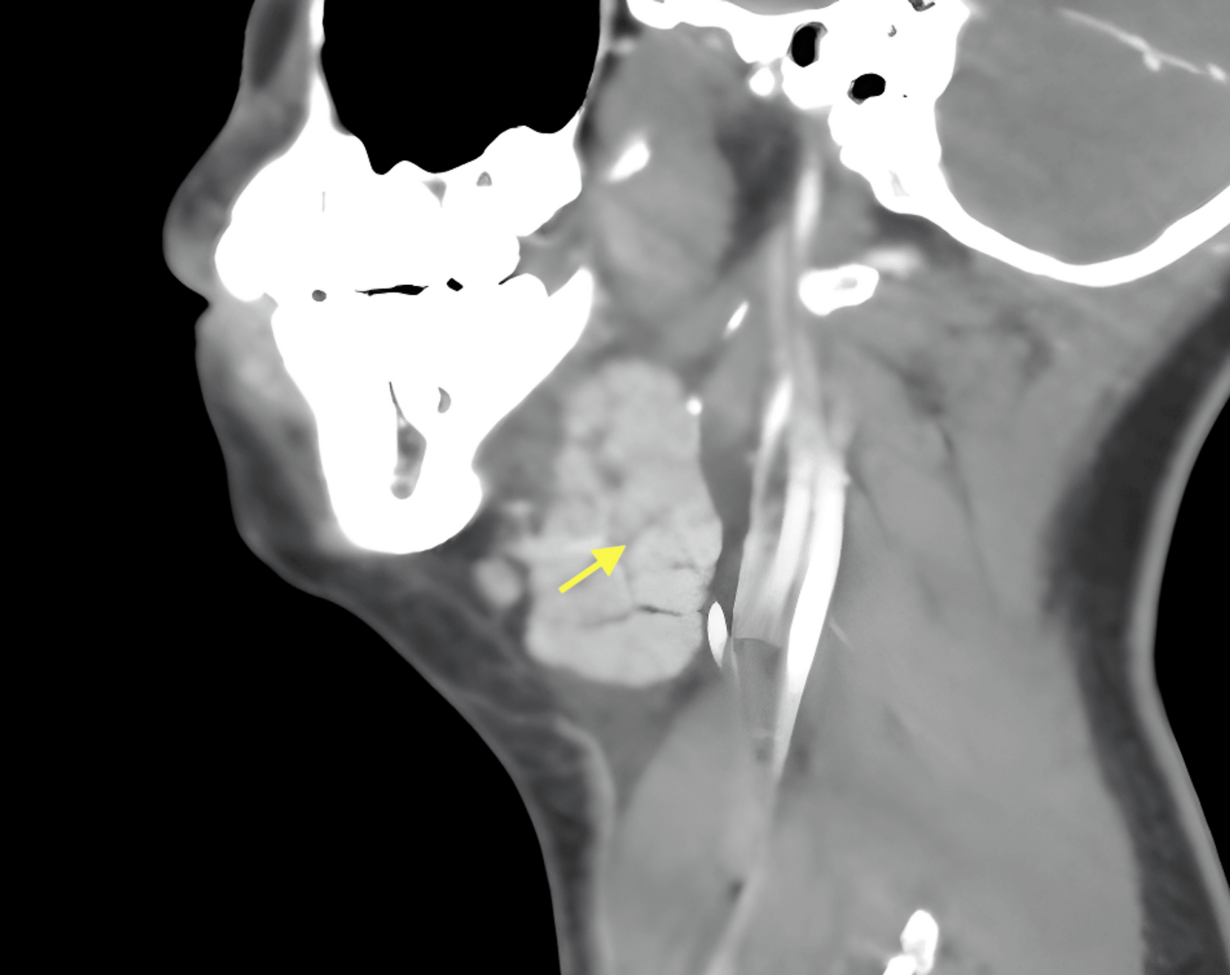
Sagittal CT neck with contrast Yellow arrow indicates the inflamed submandibular gland.

**Figure 2:**
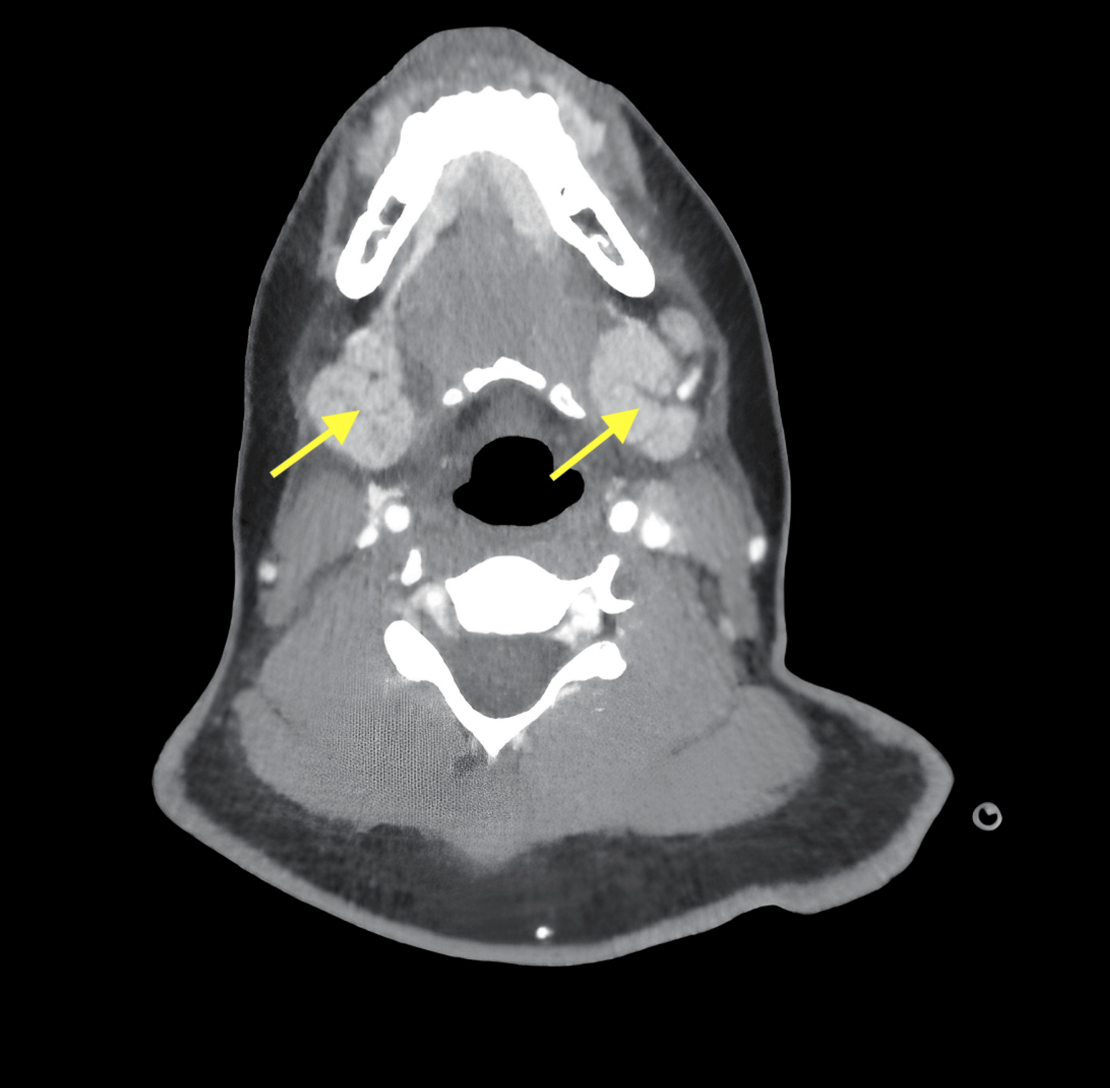
Axial CT neck with contrast Yellow arrows indicate the inflamed submandibular glands.

Basic labs were benign with no evidence of leukocytosis. Diagnostic imaging with CT of the neck with contrast was obtained and demonstrated bilateral submandibular gland sialadenitis with reactive cervical lymphadenopathy correlating with the cervical masses palpable on exam (Figures 1-2). There was no evidence of associated sialolithiasis or metastatic masses. 

The patient received a dose of intravenous methylprednisolone 125 mg and was transitioned to a rapid prednisone taper of 30 mg once daily for one day, then 20 mg once daily for one day, then 10 mg once daily for three days, and then stopped. When the patient was seen in outpatient follow-up one week after discharge, the clinical signs and symptoms of sialadenitis had completely resolved. She proceeded with immunotherapy as scheduled, without changes to the dosing, and did not experience any recurrence of symptoms.

## Discussion

Sialolithiasis, or salivary gland stones, is the most common etiology of obstructive sialadenitis and has a prevalence of about 1% in the general population [3]. The bilateral nature of our patient’s presentation and lack of obstructive mass on imaging makes sialolithiasis less likely. Acute bilateral sialadenitis is much less common and etiologies include viral, allergic, or autoimmune. Typical viral etiologies include mumps, parainfluenza, Epstein-Barr virus, and human immunodeficiency virus. The lack of systemic symptoms suggestive of infection or allergy makes these etiologies less likely. Drugs such as diuretics or anticholinergics that result in xerostomia can predispose to sialadenitis secondary to salivary stasis with retrograde contamination by bacteria of the oral cavity, but these agents were not part of our patient’s medications, and she did not report experiencing dry mouth. The most likely etiology of our patient’s sialadenitis was immunotherapy-related given the timing with recent initiation of combination ipilimumab/nivolumab therapy. 

Sicca syndrome symptoms are known to occur in approximately 6.5% of the immunotherapy treatment population [4]. While immunotherapy is known for exacerbating or unmasking underlying autoimmune conditions, immunotherapy-related sialadenitis can present as a distinct entity from Sjögren's syndrome, with no evidence of characteristic anti-Sjögren's syndrome-related antigen A (SS-A)/Sjögren's syndrome-related antigen B (SS-B) or antinuclear antibodies and with distinct salivary gland pathology on biopsy [5]. Pathology demonstrates lymphocytic infiltration as opposed to fibrosis seen in more chronic inflammatory states, and immunostaining does not suggest involvement of aquaporins or antibodies against them as seen in Sjögren's syndrome [6]. This also stands in contrast to the neutrophilic infiltration seen in suppurative bacterial infections. Post-marketing surveillance suggests that immunotherapy-related sialadenitis is extremely rare with an estimated incidence of 0.03-0.05% within the treatment population [5]. 

Distinguishing the etiology of new onset sialadenitis is critical to appropriate management. Most cases of sialadenitis will respond to conservative management. While Sjögren's syndrome is incurable and can require escalating intensity of immunomodulatory agents, immunotherapy-related sialadenitis demonstrates a robust therapeutic response to systemic corticosteroids [6].

This case could be further strengthened by gland biopsy for histology and culture, which would have characteristic lymphocytic infiltrate with no evidence of bacterial infection. Additional laboratory testing including targeted viral panel and rheumatologic and inflammatory markers would also have been informative. Given our patient’s rapid response to corticosteroids and resolution of symptoms at one week follow-up, the aforementioned studies were not justified on clinical grounds, but would have been pursued if the case did not resolve in line with the natural history of immunotherapy-related sialadenitis.

## Conclusions

In conclusion, sialadenitis is a common condition with a broad array of possible etiologies. This case highlights a single example of an immunotherapy-related adverse effect, but any organ system within the body can be affected. Immunotherapy-related sialadenitis is a rare adverse effect of immune checkpoint inhibitors, but given the ever-broadening application of these oncologic pharmaceuticals and their diverse side effect profile, clinicians must maintain an open mind when formulating a working diagnosis in these patients. Even the more common manifestations such as dermatitis or colitis can be overlooked, as they remain rare in relation to the population as a whole. Increasing awareness regarding the uses and complications related to immunotherapy will hopefully cut down on inappropriate or delayed treatment. Further research regarding optimization of corticosteroid dosing and duration, as well as the role of conservative management in this condition, would aid in the care of patients with this rare complication moving forward.
